# Feasibility of resistance training in adult McArdle patients: clinical outcomes and muscle strength and mass benefits

**DOI:** 10.3389/fnagi.2014.00334

**Published:** 2014-12-11

**Authors:** Alfredo Santalla, Diego Munguía-Izquierdo, Lidia Brea-Alejo, Itziar Pagola-Aldazábal, Jorge Díez-Bermejo, Steven J. Fleck, Ignacio Ara, Alejandro Lucia

**Affiliations:** ^1^Department of Sports Sciences, Universidad Pablo de OlavideSeville, Spain; ^2^Research Institute “i+12”, Hospital 12 de OctubreMadrid, Spain; ^3^Faculty of Sports Sciences, European UniversityMadrid, Spain; ^4^Department of Kinesiology, University of Wisconsin-Eau ClaireEau Claire, WI, USA; ^5^GENUD Toledo Research Group, University of Castilla-La ManchaToledo, Spain; ^6^School of Doctorate Studies and Research, Laboratory P-102, European UniversityMadrid, Spain

**Keywords:** rhabdomyolysis, muscle atrophy, muscle weakness, glycogenosis, weight lifting, exercise is medicine

## Abstract

We analyzed the effects of a 4-month resistance (weight lifting) training program followed by a 2-month detraining period in 7 adult McArdle patients (5 female) on: muscle mass (assessed by DXA), strength, serum *creatine kinase* (CK) activity and clinical severity. Adherence to training was ≥84% in all patients and no major contraindication or side effect was noted during the training or strength assessment sessions. The training program had a significant impact on total and lower extremities’ lean mass (*P* < 0.05 for the time effect), with mean values increasing with training by +855 g (95% confidence interval (CI): 30, 1679) and +547 g (95%CI: 116, 978), respectively, and significantly decreasing with detraining. Body fat showed no significant changes over the study period. Bench press and half-squat performance, expressed as the highest value of average muscle power (W) or force (N) in the concentric-repetition phase of both tests showed a consistent increase over the 4-month training period, and decreased with detraining. Yet muscle strength and power detraining values were significantly higher than pre-training values, indicating that a training effect was still present after detraining. Importantly, all the participants, with no exception, showed a clear gain in muscle strength after the 4-month training period, e.g., bench press: +52 W (95% CI: 13, 91); half-squat: +173 W (95% CI: 96, 251). No significant time effect (*P* > 0.05) was noted for baseline or post strength assessment values of serum CK activity, which remained essentially within the range reported in our laboratory for McArdle patients. All the patients changed to a lower severity class with training, such that none of them were in the highest disease severity class (3) after the intervention and, as such, they did not have fixed muscle weakness after training. Clinical improvements were retained, in all but one patient, after detraining, such that after detraining all patients were classed as class 1 for disease severity.

## Introduction

There is an urgent need to improve the treatment and prevention of aging sarcopenia as well as the muscle atrophy commonly associated with chronic disorders. Although several pharmacological therapies have been proposed to minimize sarcopenia and atrophy, they are not free of adverse side effects (Sanchis-Gomar et al., 2014). Physical exercise, particularly resistance (strength) exercise (i.e., movement, such as weight lifting or exercises with resistance bands, performed against a specific external force that is regularly increased during training), is an alternative approach to reverse muscle atrophy, although it is frequently overlooked in the clinical setting. Yet this type of exercise should form part of the routine exercise prescription to maintain and improve health and functional status in most, if not all population groups (Garber et al., 2011).

McArdle disease (glycogenosis (or glycogen storage disease) type V or *myophosphorylase* deficiency; OMIM® database number 232600), is an inborn disorder of skeletal-muscle carbohydrate metabolism characterized by failure of muscle glycogen breakdown. McArdle disease is a challenging disease model in which to study the feasibility and effects of resistance exercise in debilitated people: this disorder is arguably the paradigm of human exercise intolerance and rhabdomyolysis (Lucia et al., 2008). The latter is reflected by the efflux of intra-muscle proteins to the bloodstream, e.g., *creatine kinase* (CK) and myoglobin. Thus, high serum CK activity (typically > 1,000 U·L^−1^) caused by exercise is a common finding in these patients, which can be accompanied by myoglobinuria, typically referred to as “dark urine” (Santalla et al., 2014). Additionally, fixed muscle weakness is an incident problem as patients age (Santalla et al., 2014). Despite some studies showing the benefits of light-moderate intensity aerobic exercise in patients with McArdle disease (Haller et al., 2006; Maté-Muñoz et al., 2007; Perez et al., 2008), at present, clinicians in charge of these patients discourage performance of strenuous exercise, particularly resistance exercise (e.g., weight lifting), owing to a potential increased risk of severe rhabdomyolysis, which might eventually lead to acute renal failure in the most severe cases (Lucia et al., 2008). Yet preliminary data from our group indicated increases in dynamic muscle strength with no myoglobinuria in a 14-year-old male patient with McArdle disease in response to a 6-week, supervised light-moderate intensity weight lifting training program (García-Benítez et al., 2013). However, muscle mass, an important health indicator, was not determined.

We assessed the effects of a 4-month resistance training program followed by a 2-month detraining period in a group of adult McArdle patients on the following outcomes: muscle mass and strength, serum CK activity, and clinical severity.

## Methods

### Patients

Before entering the study, written informed consent was obtained from each participant, and the study was approved by the local human investigations committee and review board. Inclusion criteria were: adult with no disease contraindicating exercise other than McArdle disease, belonging to class severity 1–3 (Martinuzzi et al., 2003) (see below), living in the Madrid area (or willing to move there for the duration of the study period) to participate in all testing and training sessions. Eight McArdle patients (5 female) originally volunteered to participate in this study but one of them (male) withdrew because he had to move to a different country due to professional obligations after the first strength assessments. Genetic diagnosis was confirmed in all patients, i.e., they harbored documented pathogenic genotypes in the gene (*PYGM*) encoding *myophosphorylase* (Lucia et al., 2012), as shown in Table 1 (main demographic and clinical characteristics). All subjects reported symptoms of exercise intolerance since childhood and 4 belonged to the highest severity class, i.e., class 3 (that is, they had fixed muscle weakness) (Martinuzzi et al., 2003).

**Table 1:** **Main characteristics of the study participants at the start of the study**.

Subject	Sex	Age (years)	BMI (kg·m^2−^)	*PYGM* genotype	Diagnostic corroborated by muscle biopsy*	Resting CK (U·L^−1^)	Fixed muscle weakness	Clinical severity class
A	Female	36	18.3	*R50X/p.V456M*	Yes	1076	Yes	3
B	Female	34	30.6	*R50X/W798R*	Yes	3938	Yes	3
C	Male	23	21.7	*G205S/c.1768+1G > A*	No	1556	No	1
D	Male	29	23.7	*G205S/c.1768+1G > A*	Yes	2050	No	2
E	Female	36	21.9	*p.R50X/p.R50X*	Yes	1373	No	2
F	Female	58	27.4	*R50X/p.K754fsX49*	Yes	1211	Yes	2
G	Female	53	29.1	*G205WS/R590H*	Yes	543	Yes	3

Abbreviations: BMI, body mass index. * Negative histochemical reaction for myophosphorylase and no myophosphorylase activity.

*Note: Subjects C and D were brothers. Muscle fixed weakness affected mostly proximal/trunk (paraspinal, neck flexor, periscapular, proximal upper limb, axial or shoulder girdle) muscles in a symmetric matter in patients A, B and F, whereas it affected mostly lower extremities’ muscles (also in a symmetric manner) in patient G. See text for description of clinical severity classes*.

### Design

All study outcomes were assessed in each patient at 3 time points: before (baseline) and after the 4-month training period (“post-training”), and after detraining. Muscle strength and serum CK activity (see below) were also measured at the end of the 1st, 2nd and 3rd month of the training period. Owing to the relative rare nature of the disease (prevalence of ~1/167,000 in Spain) (Lucia et al., 2012), it was not possible to gather a sufficient number of patients to conduct a randomized controlled trial. Thus, a quasi-experimental reversal design was used, in which each subject acts as their own control (Thomas et al., 2005).

### Training program

All training sessions and strength evaluations were performed in the same setting, i.e., in the gymnasium of the *Universidad Europea* (Madrid, Spain) and were supervised by experienced professionals (fitness instructors with a Master degree in Sports Science, 1 instructor/patient). Two familiarization sessions were performed by each subject, prior to starting the training program, which included 2 weekly sessions for 4 months (total of planned sessions = 32). A recovery period of at least 48 h was allowed between sessions, and the vast majority of the sessions were performed during week days. Make-up sessions were allowed (including during weekend days) when 1 session was missed and if fulfilling the criteria of ≥48 h of recovery between sessions.

Before each training or strength assessment session (see below), patients performed 2 consecutive warm-up sessions of 12-minute duration each, the first on an arm-crank ergometer and the second on a cycle-ergometer, in order to trigger the occurrence of the “second wind” (that is, the attenuation of early fatigue, increased risk of contractures and rhabdomyolysis that commonly occurs after 7–8 min of dynamic exercise in these patients (Vissing and Haller, 2003)) in both upper and lower body muscles, respectively. The end of the warm-up was followed by ingestion of a commercialized sports drink (330 mL, containing ~30 g of sucrose). All training and strength assessment sessions were followed by passive stretching exercises and hydration with plain water. We did not perform dietary analysis but patients were instructed to consume a high proportion (65%) of complex carbohydrates (fruits, cereals, bread, pasta, rice) in the 2 meals (breakfast and lunch) that preceded each testing or training session (García-Benítez et al., 2013).

Except for the first 8 sessions of the program (where sets of 10 repetitions with very low loads were performed), the exercises composing sessions were performed for sets of a low number of repetitions (5–6) using a load (kg) eliciting a rating of perceived exertion (RPE) of 6–7 (on a 0 (= minimum effort) to 10 (= maximum effort) scale). Exercises were performed using a circuit involving large muscle groups and specific weight training equipment (Technogym; Gambetolla, Italy), in the following order: bench press, leg press, pull down and abdominals. At the end of the 1st month, the leg and bench press exercises were gradually replaced by the half squat and bench press performed on a “multipower” machine (Technogym; Gambetolla, Italy). The low number of repetitions allows the use of muscle phosphocreatine (PC) as the main energy substrate to fuel contraction, with no major reliance on muscle glycogen deposits and the circuit structure, with 2–3-min rest periods between each set of repetitions and exercises, was designed to allow PC to be resynthesized in a given muscle before this muscle was utilized again.

Passive stretching exercises were performed after each set of an exercise to attenuate muscle stiffness (3 × 30 s for each muscle group). The load was adjusted after the 1st month and thereafter was readjusted according to the results of the last strength assessment, with the purpose of reaching peak power with a similar number of sets (i.e., 4–6 sets for bench press and 6–8 sets for half-squat). The rate of increase in training load was constantly adjusted according to the patients’ RPE. Thus, when the patient reported a RPE value of 6 for a given exercise in 2 consecutive sessions, the load for this particular exercise was increased (with the premise that RPE for the new load remained ≤7). On average, the load used for an exercise increased from the start to the end of the training program as follows: 5.4 kg→12.8 kg (+139.7%) for chest press; 34.9 kg→12.7 kg (+174%) for half-squat; 10.3 kg→19.2 kg (+85.9%) for pull down; and 3.9 kg→18.3 kg (+368.1%) for abdominal muscles.

### Outcome assessment

**Figure 1:**
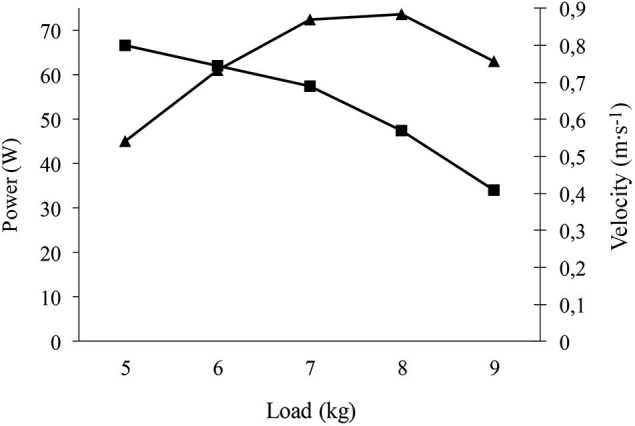
**Example of strength test (bench press) performed by one of the patients**. The Figure shows that the velocity (ms^−1^) decreases with increasing load (kg), while the average concentric-propulsive power initially increases to a load in which the decrease of velocity is so pronounced that power decreases. The highest average concentric-propulsive power of 73 W occurred at a load of 8 kg.

After familiarization with the equipment, participants performed “explosive” leg half-squats on a “Multipower machine”, which was connected to a linear encoder (T-Force Dynamic Measurement System, Ergotech, Murcia, Spain). The latter has previously proven valid to determine force (N) and power (W) (González-Badillo and Sánchez-Medina, 2010). Patients performed 1 set of 3 repetitions at maximum speed with a 2-min recovery period. The load or “resistance” (kg) was increased by 2.5 kg in each successive set. Average muscle force and power output in the concentric-propulsive phase of the repetition were evaluated in each set (Sanchez-Medina et al., 2010). In this type of gradual resistance increase protocol, the developed muscle force increases with resistance, while the velocity of muscle contraction decreases. Power is the product of force × velocity, and initially increases with resistance and then decreases when the resistance causes a substantial decrease in velocity. Thus, the test is stopped when the decrease in velocity is so pronounced that it causes a decrease in average muscle concentric power (see Figure 1 for an example). For statistical analyses we recorded the highest value of average power (W) in the concentric-propulsive phase, which typically coincides with the start of a decline in this variable together with the occurrence of the highest value of average force (N). We also recorded the load (kg) at which maximum average power was generated. The RPE after each set never exceeded the value of 7.

Total and regional body composition was assessed by dual energy x-ray absorptiometry (DXA; Hologic Serie Discovery QDR, Software Physician’s Viewer, APEX System Software Version 3.1.2. Bedford, MA, USA). Body mass composition was calculated from whole-body scans. Whole-body scans were submitted to a regional analysis to determine the composition of the arm, leg and trunk regions. The arm region included the hand, forearm and arm, and was separated from the trunk by an inclined line crossing the scapulo-humeral joint, such that the humeral head was located in the arm region. The leg region included the foot, the lower leg and the upper leg. It was separated from the trunk by an inclined line passing just below the pelvis, which crossed the neck of the femur. The trunk region included the entire body except the arms, legs and head regions. The head region comprised all skeletal parts of the skull and cervical vertebra above a horizontal line passing just below the jawbone. With this analysis, regional body fat and lean mass can be assessed with a coefficient of variation below 5% (Calbet et al., 1998).

Peripheral venous blood was collected from all subjects to determine serum total CK activity, a widely used marker of skeletal muscle damage (Sorichter et al., 1999). CK activity was determined using a standard photometric analyzer (Hitachi 911, Boehringer Mannheim, Mannheim, Germany), at baseline (under “resting” conditions, that is, after 24 or more hours with physical activities restricted to the minimum) at the following time points: pre-training, before each strength assessment (end of 1st, 2nd, 3rd and 4th month), and after detraining. Serum total CK activity was also measured 1 h after each strength assessment (end of 1st, 2nd, 3rd and 4th month). The mean baseline values in our laboratory for adult male and female McArdle patients are 3,069 ± 2,356 and 1686.2 ± 1964.8 U·L^−1^, respectively, whereas the mean baseline values for healthy aged-matched controls are 151 ± 48 and 97.7 ± 35.7 U·L^−1^, respectively.

Identification of patient clinical features allowed us to allocate them to one of the following clinical severity classes according to the most commonly used phenotype severity scale (Martinuzzi et al., 2003): “0 = asymptomatic or virtually asymptomatic (mild exercise intolerance, but no functional limitation in any daily life activity); 1 = exercise intolerance, contractures, myalgia, and limitation of acute strenuous exercise, and occasionally in daily life activities; no record of myoglobinuria, no muscle wasting or weakness; 2 = same as 1, plus recurrent exertional myoglobinuria, moderate restriction in exercise, and limitation in daily life activities; 3 = same as 2, plus fixed muscle weakness, with or without wasting and severe limitations on exercise and most daily life activities”.

### Statistical analysis

In order to decrease the risk of statistical type I error, we used the nonparametric Friedman test (instead of a repeated-measures ANOVA) to compare within subjects the mean values of all the variables measured at the different time points. All statistical tests were performed using the Social Sciences package (SPSS, 2010, IBM SPSS Statistics 19 Core System User’s Guide; SPSS Inc., Chicago, IL). Also to avoid type I error, *post hoc* pairwise comparisons were only performed when a significant time effect was found. Significance was set at *α* = 0.05 and results are expressed as means ± standard error of the mean (SEM).

## Results

### Adherence and side effects

Adherence to training was 100% in 5 patients and 84% in the 2 remaining patients (with reasons for missing sessions being independent from the training itself, i.e., viral respiratory infection in 1 patient and household or children care tasks in the other one). No major contraindication was noted during the training or strength assessment sessions other than the usual muscle discomfort and soreness associated with resistance exercise in non-habituated people (especially during the initial sessions). We only had to interrupt a given set of repetitions due to muscle stiffness on 5 occasions (2 patients). No episode of myoglobinuria (i.e., no occurrence of “dark urine”) was reported, which is consistent with the fact that serum CK activity levels showed no major increases above the reference limits for this population (see below).

The training program had a significant impact on total and lower extremities’ lean mass (*P* < 0.05 for the time effect), with mean values increasing with training and decreasing with detraining (Table 2). In contrast, body fat mass remained essentially unchanged (*P* > 0.05). The increase in total or lower extremities’ lean mass from pre- to post-training averaged +855 g (95% confidence interval (CI): 30, 1679) and +547 g (95% CI: 116, 978), respectively, with all patients showing an increase with training, except one patient (i.e., patient B, 33 years, severity class 3). Detraining resulted in a significant decrease in total (−1,222 g (95%CI: −2,585, 340)) and lower extremities’ lean mass compared to post-training (−521 g (95%CI: −846, −197)). Detraining values of total and lower extremities’ lean mass were not significantly different from pre-training values.

**Table 2:** **Mean ± SEM values of body composition assessed by dual energy x-ray absorptiometry (DXA)**.

Outcome	Pre-training	Post-training	Detraining	Time effect	Pre- vs. post-training	Pre- vs. detraining	Post- vs. detraining
**Lean mass**
Total (g)	43,089 ± 1,997	43,944 ± 1,935	42,822 ± 2,099	***P* = 0.018**	***P* = 0.043**	*P* = 0.917	***P* = 0.018**
Trunk (g)	21,179 ± 869	21,378 ± 833	20,928 ± 882	*P* = 0.772	-	-	-
Legs (g)	14,662 ± 825	15,209 ± 815	14,688 ± 889	***P* = 0.018**	***P* = 0.043**	*P* = 0.753	***P* = 0.018**
Arms (g)	4,110 ± 353	4,206 ± 381	4,008 ± 398	*P* = 0.121	-	-	-
**Body fat (g)**	21,571 ± 2,880	21,473 ± 2,896	21,009 ± 2,625	***P* = 0.368**	-	-	-

*Significant P-values are in bold. Pairwise post hoc comparisons (Wilcoxon test) were only performed when a significant time effect was found*.

**Figure 2:**
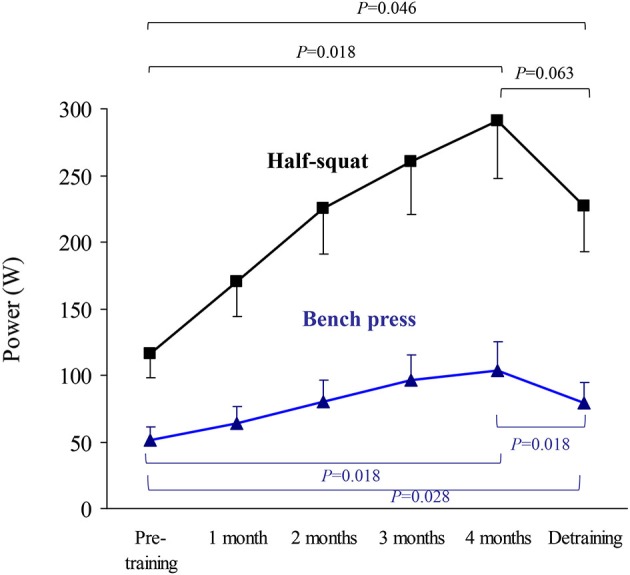
**Results (mean ± SEM) of upper body (bench press) and lower body muscle strength (half-squat) expressed as highest value of average muscle power in the concentric-propulsive repetition phase**. Significant differences between time points are indicated by brackets. Of note, due to the high number of time points during the training period, and thus to minimize type I error, pairwise *post hoc* comparisons of pre vs. post-training were only performed at the end (4th month) of the training period.

**Figure 3:**
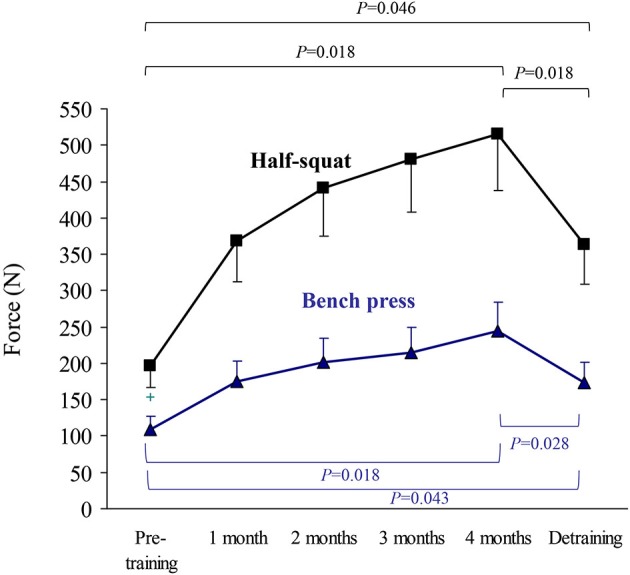
**Results (mean ± SEM) of upper body (bench press) and lower body muscle strength (half-squat) expressed as highest value of muscle force (N) in the concentric-propulsive repetition phase**. Significant differences between time points are indicated by brackets. Of note, due to the high number of time points during the training period, and thus to minimize type I error, pairwise *post hoc* comparisons of pre- vs. post-training were only performed at the end (4th month) of the training period.

**Figure 4:**
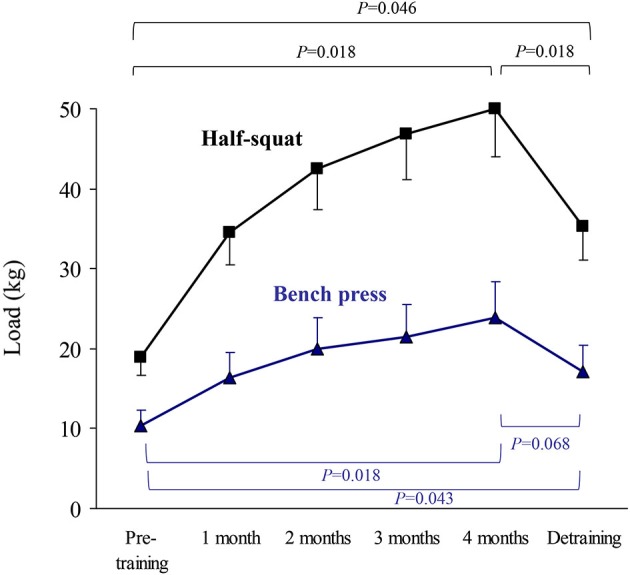
**Results (mean ± SEM) of upper body (bench press) or lower body muscle strength (half-squat) expressed as the load eliciting the highest value of average muscle power in the concentric-propulsive repetition phase**. Significant differences between time points are indicated by brackets. Of note, due to the high number of time points during the training period, and thus to minimize type I error, pairwise *post hoc* comparisons of pre- vs. post-training were only performed at the end (4th month) of the training period.

The results of upper body (bench press) or lower body muscle strength (half-squat), expressed as the highest value of average muscle power (W, Figure 2) or force (N) in the concentric-propulsive phase of repetitions (Figure 3), or as the load (kg) eliciting such values (Figure 4), showed the following overall pattern: consistent increase (e.g., bench press: +52 W (95% CI: 13, 91): half-squat: +173 W (95% CI: 96, 251)) over the 4-month training period (such that post-training values were significantly higher than pre-training values), and a decline after detraining. Detraining resulted in a significant loss compared to post-training, but strength after detraining was still significantly greater than at pre-training. Importantly, all the participants, with no exception, showed a clear gain in muscle strength after the 4-month training period.

**Figure 5:**
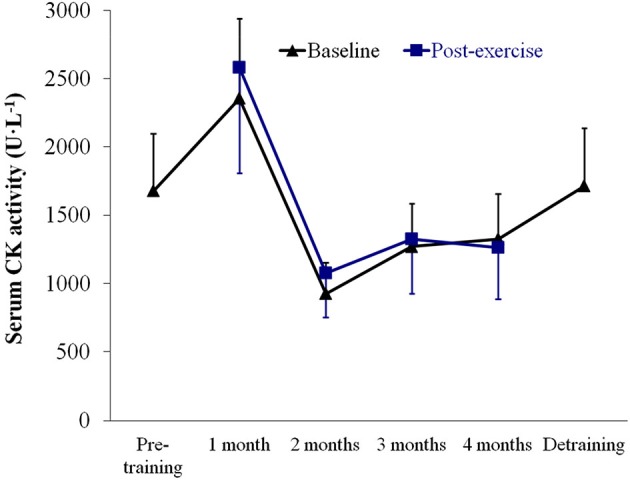
**Results (mean ± SEM) of serum *creatine kinase* (CK) activity assessed at baseline (resting conditions) and 1 h after the strength tests**. No significant time effect (*P* > 0.05) was noted for baseline or post-strength assessment values.

No significant time effect (*P* > 0.05) was noted for baseline or post-strength assessment values of serum CK activity (Figure 5), which remained essentially within the range values reported in our laboratory for McArdle patients (and well below the upper limit value of 95% CI (3,387 U·L^−1^)), indicating that the training program did not induce major increases in CK-emia. Further, an increasing trend in CK levels was noted in the 1st month of training that was reversed thereafter, which reflected a positive adaptation to the program.

**Figure 6:**
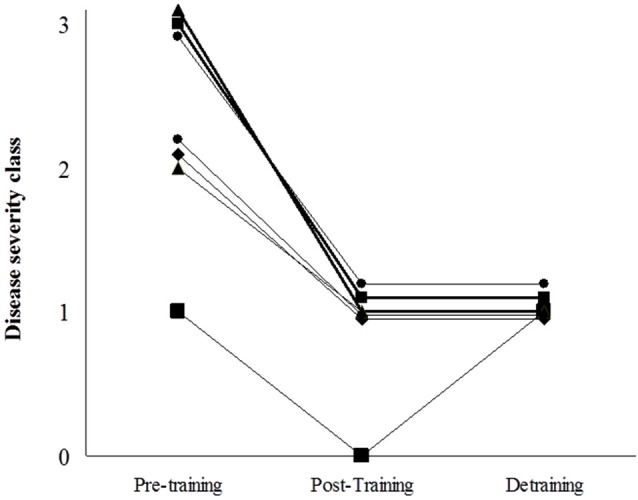
**Patients’ individual scores of disease severity using the scale by Martinuzzi et al (Martinuzzi et al., 2003): “class 0 = asymptomatic or virtually asymptomatic (mild exercise intolerance, but no functional limitation in any daily life activity); 1 = exercise intolerance, contractures, myalgia, and limitation of acute strenuous exercise, and occasionally in daily life activities; no record of myoglobinuria, no muscle wasting or weakness; 2 = same as 1, plus recurrent exertional myoglobinuria, moderate restriction in exercise, and limitation in daily life activities; 3 = same as 2, plus fixed muscle weakness, with or without wasting and severe limitations on exercise and most daily life activities”**.

The clinical course of the disease is shown in Figure 6. All patients changed to a lower severity class with training, such that none of them belonged to the highest disease severity category (class 3) after training and, as such, did not have fixed muscle weakness anymore. Further, 2 patients moved to class 0, which is essentially symptom-free. Most of the clinical improvements (except for one patient) were retained at detraining, that is, all patients were classed as class 1 for disease severity.

## Discussion

The main, novel findings of our study were threefold. First, the training program was feasible, free of noticeable adverse effects (with no episode of myoglobinuria and with CK-emia remaining within safe limits) and well tolerated by the patients. This is especially relevant when considering that ~60% of them originally belonged to the highest severity class and thus had fixed muscle weakness and limitations during daily living activities such as house-hold tasks. Second, the training program significantly increased total and lower extremities’ lean mass, with all the patients except one showing an improvement in these variables. This increase in lean mass was reflected in the consistent increase (>twofold) of upper and lower body strength over the 4-month training period, with *all* the patients showing an improvement. Importantly, training-induced gains were not totally lost with detraining. Finally, the program had direct clinical benefits, because clinical severity decreased with training. Notably, after training, all the patients moved to severity classes 0–1, that is, they became virtually asymptomatic (class 0) or at most had occasional limitations in daily life activities (class 1). In addition, the improvements in clinical course were essentially retained at detraining, which is consistent with the fact that previous gains in muscle strength were not completely lost after detraining. To date, we are not aware of any therapeutic intervention showing such practical benefits in McArdle patients, and arguably of any other lifestyle intervention able to induce such clinical improvement in genetic neuromuscular disorders. No significant beneficial effects have been reported in McArdle patients receiving branched chain amino acids (MacLean et al., 1998), depot glucagon (Day and Mastaglia, 1985), dantrolene sodium (Poels et al., 1990), verapamil (Lane et al., 1986), vitamin B6 (Phoenix et al., 1998) (except in one recent case report (Sato et al., 2012)), or high-dose oral ribose (Steele et al., 1996). More controversial are the effects of creatine supplementation: low-dose supplementation (60 mg^.^kg^−1.^day^−1^ for 4 weeks) attenuated muscle complaints in 5 out of the 9 McArdle patients (Vorgerd et al., 2000) but higher doses (150 mg^.^kg^−1.^day^−1^) actually exacerbated exercise-induced myalgia for unknown reasons (Vorgerd et al., 2002). A 12-week treatment with the angiotensin converting enzyme (ACE) inhibitor ramipril (2.5 mg^.^day^−1^) attenuated disability in McArdle patients, but the effect was more marked in those harboring the D/D genotype of the insertion(I)/deletion(D) polymorphism in the *ACE* gene (Martinuzzi et al., 2008). A short-term trial (10 days) with a “read through” compound able to synthesize full proteins from transcripts containing premature termination stop codons (i.e., gentamicin) failed to normalize 31P magnetic resonance spectroscopy indicators of *myophosphorylase* deficiency in the muscle of McArdle patients (Schroers et al., 2006).

This is the first report on the feasibility and functional and clinical effects of a resistance training program in adult McArdle patients. The data are novel and we believe that assessing the applicability of this type of program in adults with McArdle disease is of medical interest because this type of exercise should form part of the routine exercise prescription to maintain and improve health and functional status in most, if not all population groups (Garber et al., 2011). Resistance training is also gaining growing attention for its effectiveness in attenuating aging sarcopenia as well as the status of muscle weakness and atrophy that accompanies most chronic conditions, such as neuromuscular disorders (Sanchis-Gomar et al., 2014). This is an important consideration also in McArdle disease because the baseline values of muscle mass and strength of the study participants were quite low compared to the general population. For instance, the mean total lean mass (~42 kg) of the 5 female patients (age range: 34–48 years) at the start of the program, was only ~6% higher than the values recently reported in old, sedentary Spanish women (mean age 75 years) (Gómez-Cabello et al., 2013a), and the values of upper body lean mass were even lower (~−11%) in the same McArdle patients compared to the old sedentary Spanish women (Gómez-Cabello et al., 2013b). The latter finding is consistent with the fact that 4 of the female patients had fixed muscle weakness affecting mostly proximal muscles. As for the muscle strength values, recent research with the same test and equipment for strength assessment used here showed that young male adults with a mean age comparable to that of the 2 studied male patients had average values of peak power during the bench press test (at 30 kg) of ~320 W (vs. only 140–188 W at the same load in our male patients) (Sanchez-Medina et al., 2010).

Concerns are frequently raised by clinicians as to the potential risks of exercise, particularly weight lifting, in McArdle patients. However, serum CK activity did not increase with resistance training (and in fact tended to decrease with training after the 1st month, indicating a good muscle tissue adaptation to the program) and we recorded no incidence of myoglobinuria. This indicates that the weight training program was well tolerated. On the other hand, it must be kept in mind that muscle damage, or at least some degree of it, as indicated by high serum CK activity, is a necessary physiological stimulus for muscle to be repaired and adaptative hypertrophy to occur (Clarkson and Hubal, 2002). Our results are consistent with those of a previous case report study from our laboratory showing the functional benefits of resistance training in an adolescent with McArdle disease (García-Benítez et al., 2013). Muscle mass was not measured in this case study and thus it could not be determined to what extent muscle strength gains were due to neuromuscular adaptations only or to the occurrence of some degree of muscle hypertrophy, whereas here we showed that exercise-induced hypertrophy is an attainable goal in McArdle patients. The finding that both muscle mass and strength increased in these patients is of clinical relevance because low muscle mass and poor muscle strength are highly prevalent among westerners and are important risk factors for disability and potentially mortality in individuals as they age (especially if combined with high adiposity), whereas high muscle mass and strength are associated with a healthier cardiometabolic phenotype (Kalyani et al., 2012). With regards to this, besides the problem of the low muscle mass/strength levels shown here, recent research from our group has indicated that McArdle patients have an overall unfavorable cardiometabolic profile (Munguía-Izquierdo et al., 2014). On the other hand, the fact that gains in muscle strength were not totally lost after detraining is in overall agreement with previous research in healthy adults (Mujika and Padilla, 2001) or in chronic disease populations (Herrero et al., 2006) showing that, at least compared to muscle oxidative capacity, muscular strength suffers a more limited decrease after relatively short periods of detraining. This phenomenon is due, at least partly, to the fact that gains in neuromuscular performance (i.e., motor unit recruitment) can be relatively retained during periods of detraining (Mujika and Padilla, 2001).

Our study is not without limitations. First, we did not assess a control group of McArdle patients receiving no exercise intervention, although the quasi-experimental design we used (where each subject acted as their own control) might overcome, at least partly, this limitation. It would have been interesting to compare the effects of the present resistance intervention with other types of exercise programs (e.g., stair climbing, brisk walking) in McArdle patients. On the other hand, assessing healthy controls performing the same weight training intervention might had allowed us to determine if the ability to gain muscle mass and strength is limited (or not) in McArdle patients compared to non-patients. Finally, further research might determine if adding protein or creatine supplements to the current training program might contribute to maximize the gains in patients’ muscle mass.

In summary, if appropriate training guidelines are followed (i.e., qualified instruction, competent supervision, and appropriate progression of the volume and intensity of training as we did here), regular participation in a strength training program has the potential to improve the muscle strength and mass, as well as the clinical status, of McArdle patients. While keeping in mind the need for large sample intervention studies (which might not be easily feasible in rare diseases as this one), our preliminary data suggest that supervised resistance training is feasible in McArdle patients and has medical benefits, i.e., increased muscle strength and force and attenuation of clinical severity. Thus, we believe that the statement that “exercise is medicine” also applies to a disease which has been traditionally considered to be the paradigm of exercise intolerance, especially with regard to weight lifting.

## Conflict of interest statement

The authors declare that the research was conducted in the absence of any commercial or financial relationships that could be construed as a potential conflict of interest.
